# Biophysical and Integrative Characterization of Protein Intrinsic Disorder as a Prime Target for Drug Discovery

**DOI:** 10.3390/biom13030530

**Published:** 2023-03-14

**Authors:** Shuqi Luo, Samuel Wohl, Wenwei Zheng, Sichun Yang

**Affiliations:** 1 Center for Proteomics and Department of Nutrition, School of Medicine, Case Western Reserve University, Cleveland, OH 44106, USA; 2 Department of Physics, Arizona State University, Tempe, AZ 85287, USA; 3 College of Integrative Sciences and Arts, Arizona State University, Mesa, AZ 85212, USA; 4 Case Comprehensive Cancer Center, Case Western Reserve University, Cleveland, OH 44106, USA

**Keywords:** protein intrinsic disorder, integrative biophysics, drug discovery

## Abstract

Protein intrinsic disorder is increasingly recognized for its biological and disease-driven functions. However, it represents significant challenges for biophysical studies due to its high conformational flexibility. In addressing these challenges, we highlight the complementary and distinct capabilities of a range of experimental and computational methods and further describe integrative strategies available for combining these techniques. Integrative biophysics methods provide valuable insights into the sequence–structure–function relationship of disordered proteins, setting the stage for protein intrinsic disorder to become a promising target for drug discovery. Finally, we briefly summarize recent advances in the development of new small molecule inhibitors targeting the disordered N-terminal domains of three vital transcription factors.

## 1. Introduction

Intrinsic disorder in proteins is becoming important due to its prevalence in the human proteome and its roles in cellular signaling in normal and abnormal cells [1]. The amino acid sequence of these intrinsically disordered proteins (IDPs) presents a challenge, as it lacks a well-defined 3D structure and is highly flexible. The demand for their functional and disease-driven understanding is beyond simple sequence-based bioinformatic analysis. An in-depth understanding requires adding the “structure” component to the disorder–function relationship typically expected for structurally folded proteins.

The high flexibility of IDPs prompts a revisit of available biophysical tools. For simplicity, we categorize these tools into experimental and computational methods before discussing their synergistic integration. These techniques often complement one another, fostering the growth of integrative biophysics through a combination of experiments and computations.

The biophysical studies of IDPs present opportunities for their potential application in drug discovery due to their links to various diseases. Targeting the disorder itself, rather than its upstream or downstream coregulator proteins, has been found to be viable, with multiple successful examples reported. This short review concludes by providing an overview of the status of targeting the intrinsic disorder in the N-terminal domains (NTDs) of three key transcription factors: p53, androgen receptor (AR), and estrogen receptor (ER).

## 2. Experimental Biophysical Techniques

**Figure 1:**
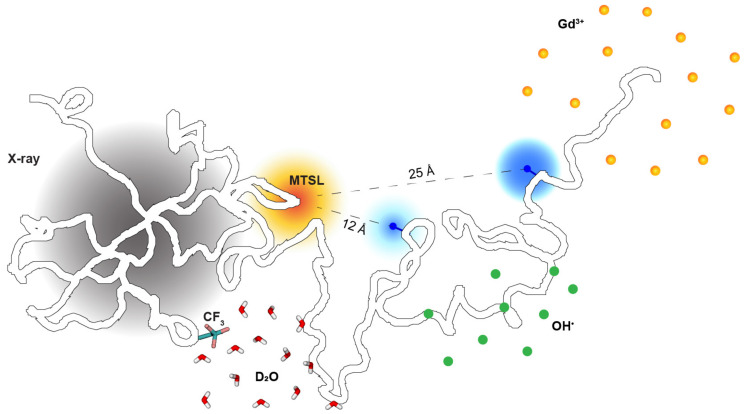
A schematic diagram of various biophysical techniques probing the structural properties of intrinsically disordered proteins (IDPs). A large gray circle illustrates the 2D pattern from small-angle X-ray scattering to provide information about the global conformation and the pairwise distance distribution between atoms within the protein. MTSL (large orange circle): a nitroxide spin label; blue dots: observed NMR-active nuclei whose signal intensity is attenuated (blue circles) to monitor specific distances from the paramagnetic spin-label, typically within the range of 12–25 Å. Gd^3+^ represents gadodiamide. OH: hydroxyl radicals; CF_3_: a trifluoromethyl tag; D_2_O: heavy/deuterated water. These agents act as a probe to detect site-specific information about solvent accessibility at the peptide or single-residue level.

IDPs vary in molecular size from tens to over one thousand amino acids weighing more than 100 kDa [2]. Each IDP may require different biophysical techniques for structural analysis due to varying chain lengths. Nuclear magnetic resonance (NMR) spectroscopy is particularly useful for studying IDPs but is limited to small proteins, e.g., those under 25 kDa. Some other techniques have no size limit, making them suitable for larger proteins, but they may not provide the same level of detail and amino acid coverage as NMR. As such, we begin this section with general techniques before discussing NMR-specific tools, some of which are illustrated in Figure 1.

### 2.1. Global Conformations via Small-Angle X-ray Scattering (SAXS)

SAXS has been a primary tool for studying the relationship between the overall radius of gyration (*R*_g_) and polypeptide length N (i.e., number of amino acids) for a broad range of unfolded and natively disordered proteins without size limits [3,4]. A power law typically describes this relationship as *R*_g_~*N^v^*, where *v* is a scaling component [5]. Exceptions may occur, particularly for proteins with a high percentage of hydrophobic amino acids [6,7], although this power law remains a proper first-order estimate from a polymer perspective.

The two critical parameters in analyzing an experimental SAXS intensity profile *I*(*q*) are the physical *R*_g_ and *v*, where *q* is the X-ray scattering distance in the reciprocal space (i.e., the amplitude of momenta transfer during the scattering). *R*_g_ is typically determined from the low-*q* region (e.g., *q∙R*_g_ < 1.3), while *v* is calculated from the high-q region (e.g., *q∙R*_g_~3–10 [8,9]). The *v* value of an IDP can range from 0.45 (for compact disorder) to 0.5 (for modest disorder) to 0.65 (for expanded disorder) [10]. This range of disorder behaviors is often illustrated in a Kratky plot of *q*^2^*∙I*(*q*) vs. *q∙R*_g_, distinguishing folded proteins with a bell-like shape from IDPs that level off to reach a plateau at high-*q* regions [9]. Other parameters, such as the Porod volume that integrates over the entire *q* region, can assist in, e.g., processing raw scattering data, but their full utilization has yet to be fully explored [11,12].

Individual IDPs are known for their high flexibility with a range/ensemble of conformations in solution. SAXS provides an ensemble-averaged representation of the distribution of distances between all pairs of atoms, a well-known approach for non-biological systems [13]. For proteins, by using the GNOM method to transform the entire *I*(*q*) profile, the pair distance distribution function can be determined [14], providing a global view of IDP conformations in aqueous equilibrium.

The high flexibility of IDPs can pose challenges for accurate SAXS data acquisition, as they tend to aggregate or show heterogeneity. Size-exclusion chromatography-coupled SAXS (SEC-SAXS) is a step-forward solution to eliminate unwanted species that may be present in the standard flow cell setup [15,16]. However, a higher protein concentration is required for SEC-SAXS due to dilution from SEC elution. With improvements in synchrotron light sources and increased X-ray brightness, protein concentration is becoming less of a concern, and the desire for accuracy and reliability is often given priority. Thus, SEC-SAXS is the preferred method for obtaining accurate scattering information in biological applications when feasible.

### 2.2. Site-Specific Solvent Accessibility through the Lens of Three Labeling Techniques

Several biophysical techniques are available to probe the solvent exposure of specific residues in a polypeptide chain. These methods typically involve labeling, quantification, and structural mapping. To show their common features and differences, we describe three exemplary techniques: H/D exchange (HDX) [17,18,19], hydroxyl radical protein footprinting (HRPF) [20,21,22,23,24,25], and D_2_O-induced fluorine chemical shift perturbations (DFCS) [6,26,27]. These labeling techniques are particularly useful for studying large proteins that NMR cannot analyze.

HDX and HRPF share similar concepts but have distinct features. HDX utilizes excess deuterated (D_2_O) buffer to exchange amide hydrogens, while HRPF relies on X-ray hydrolysis [20,28] or laser photolysis [22,29] to generate hydroxyl radicals that can irreversibly and covalently modify the sidechains of individual amino acids. The efficiency of HRPF labeling is based on the diffusion of labeling agents within a short time frame (e.g., milliseconds). The labeling site is a key difference between the methods: HDX focuses on backbone amino hydrogens and HRPF on sidechains. Both techniques often involve a dose–response process at different time windows, followed by the quenching of exchange or reaction before protein digestion by proteases into small peptic peptides.

The DFCS technique uses fluorine labeling by attaching a trifluoromethyl (–CF_3_) tag, typically from 3-Bromo-1,1,1-trifluoroacetone (BTFA), to cysteine sidechains [30,31,32,33,34]. This method is beneficial for proteins without native cysteine residues, as the tag can be placed at any position that is mutated to cysteine. However, it can be nontrivial for proteins with multiple cysteines, as it requires identifying individual labeling sites. The process can also be labor-intensive and time-consuming if multiple sites are needed for individual characterization. Each site needs a new protein construct with a cysteine mutation, as we demonstrated for 12 sites of fluorine labeling [6]. Because isotopic D_2_O water causes a change in fluorine chemical shift (up to 0.2 ppm), the fluorine tag acts as a probe to evaluate its local solvent environment at varying D_2_O concentrations.

Labeling quantification is conducted using liquid chromatography coupled with tandem mass spectrometry for HRPF and most HDX experiments, typically at the level of peptides. Advances in HRPF have enabled a single-residue description, taking advantage of the hydrodynamic difference between individual labeled sites separated by chromatography elution [35,36]. Furthermore, sample delivery has been improved using a liquid injection jet without a container [37]. Other advanced options include time-resolved HRPF, either using a rapid-mixing stopped-flow system [38,39] or a rapid-relaxation temperature jump setup [40], which has been demonstrated to study the kinetics of protein–protein binding, e.g., at the (sub-)millisecond or even microsecond timescale, providing information beyond the ensemble-averaged thermodynamic properties afforded by standard HRPF measurements. DFCS quantification is more straightforward and involves recording fluorine chemical shift spectra and identifying fluorine peaks. The rate/slope of these peaks changes as a function of D_2_O concentration report information on the exposure of the fluorine-tagged site to deuterated solvent.

Structural mapping can be achieved from the HDX rate, HRPF rate, and DFCS slope. The protection factor (PF) method has been well established for structural analysis using the HDX rate [18]. A similar PF analysis has been introduced for the HRPF rate, which accounts for variations among different amino acid types in their intrinsic rate at a peptide or single-residue level [41]. Unlike HDX and HRPF, the DFCS slope allows direct comparison between various labeling sites, utilizing the same fluorine tag uniformly [42]. 

The final amino acid position coverage varies among techniques due to labeling efficiency, location of sites, and protease digestion. In the case of a 184-residue protein [6], the HDX data provide excellent coverage at the peptide level. However, due to high solvent exposure, the averaging-out across all amino acids within each peptide cannot yield a meaningful description. In contrast, HRPF effectively characterizes the solvent exposure of 16 amino acids (out of 184), demonstrating that some residues are well protected from the solvent despite the intrinsic disorder [6].

### 2.3. Probing Single Pairwise Distances between Amino Acids 

The distance between a specific pair of amino acids can be probed via amino acid labeling. These methods include Förster resonance energy transfer (FRET) [43], double electron–electron resonance (DEER) [44,45], and photoinduced electron transfer (PET) [46]. The major difference between these distance-related methods is the relation between the experimental signal and the distance between the pair of labeled/specific amino acids. Different methods are often most sensitive to different distance regimes. Therefore, they are often applied in other contexts but can sometimes be complementary, considering the wide distance distribution between two amino acids within a conformational ensemble of an IDP. However, interpreting the physical variables from these methods can be non-trivial due to the heterogenous conformations in IDP ensembles.

FRET. The FRET method covalently links a pair of a donor and an acceptor dye at a specific amino acid site of interest along the chain. The donor dye is optically excited, and the excited energy can either be emitted as a photon or transferred to an acceptor dye. The energy transfer efficiency E is related to the distance *r* between the pair of dyes if the dye can rapidly experience different orientations within time scales of the donor lifetime. The physical interpretation of the FRET signal can be captured by the Förster equation $E\left(r\right)={\left[1+{\left(r/R_{0}\right)}^{6}\right]}^{-1}$, where *R_0_* is the Förster radius [47], a value intrinsic to a given set of dyes. This value determines the optimal distance range for FRET measurements. By varying the type of dye, *R_0_* can range from approximately 40 to 70 Å. Such a distance regime reasonably covers the averaging end-to-end distance of a 100-residue IDP with a size close to a random coil. If multiple pair labeling positions are affordable, FRET can also provide distances between more than one pair of amino acids [10,48]. This information sheds light on the conformational tendencies of various regions of an IDP and reveals scaling behavior [10] and heteropolymeric properties [49].

DEER. The DEER method, a type of electron paramagnetic resonance (EPR) spectroscopy, measures the dipole–dipole couplings between two unpaired electron spins. The spin labels can be introduced as labels on specific amino acids far apart in the sequence. DEER measurements have a distance dependence of r^−3^ in contrast to the r^−6^ dependence in FRET and are most sensitive to distances of 20–80 Å [44]. More specifically, the distance distribution can be obtained through methods such as Tikhonov regularization [50].

PET. In contrast, the PET method does not require a label attached to a specific amino acid; instead, the quenching happens between two naturally occurring amino acids, tryptophan and cysteine [46,51]. These two amino acids are not commonly seen in an IDP sequence, suggesting that it is often impossible to directly measure an IDP’s conformation without mutations. PET studies often involve mutating an aromatic amino acid to tryptophan and serine to cysteine, which minimizes the modification impact [52]. The rate of PET decays exponentially as a function of the distance between two amino acids, typically less than 8 Å [53]. This indicates that if PET is applied alone for an IDP, the sequence separation between two amino acids of interest should not exceed 40 residues. This restraint poses a challenge for the PET application, considering the typical length of an IDP. However, due to the growing interest in capturing transient specific interactions within IDPs, PET could be an alternative method focusing on these short-range distances of interest. For example, PET studies of p53-NTD have revealed a kinetic slowdown of long-range loop closure between two amino acids (e.g., V31 and W53) due to phosphorylation [54].

### 2.4. Versatile NMR Techniques

For proteins amenable to chemical shift assignments, NMR is a premier tool for in-depth investigations beyond analyzing the transient secondary structure and chemical shift perturbation [55,56,57,58]. High protein concentrations, typically above 200 uM, are required for resonance assignments. However, for 2D NMR spectra such as heteronuclear single-quantum coherence (HSQC), a lower concentration of around 30–100 uM is generally sufficient to produce adequate signal-to-noise ratios in a reasonable acquisition time, enabling its broad application to IDPs.

The sampling temperature for NMR data acquisition is an important distinction between disordered and folded proteins. For folded proteins, higher temperatures (e.g., room temperature) are commonly used to enable fast rotational diffusion for sharp resonances due to the restricted mobility of structured regions. On the other hand, lower temperatures (e.g., 4–10 °C) are favored for high-quality 2D NMR spectra of highly flexible IDPs because of the increase in line broadening caused by amide hydrogen exchange with the solvent, particularly for solvent-exposed residues.

The low protein concentration requirement for 2D NMR spectra (e.g., HSQC) is a crucial advantage in the studies of highly flexible IDPs. The reduced concentration minimizes interference from nonspecific intermolecular interactions and enables the focus on intramolecular dynamics. Furthermore, this allows using a wide range of NMR techniques to study IDPs. The most informative NMR experiments for IDPs include assessments of backbone dynamics using relaxation measurements, long-range interactions using paramagnetic relaxation enhancements (PRE), and backbone solvent accessibility using solvent-PRE. 

^15^N relaxation. Backbone dynamics can be probed through ^15^N relaxation measurements (longitudinal R_1_ and transverse R_2_) by monitoring the intensity decays of individual amino acids [59,60]. This method has been used to study both unfolded and disordered proteins. One approach uses R_1ρ_, the ^15^N longitudinal relaxation rate in the rotating frame, with longer relaxation delays to account for the relatively slow ^15^N-relaxation of disordered protein [61]. Applications include identifying residual structural features, such as hydrophobic clustering, and locating regions of restricted backbone mobility as indicated by large R_2_/R_1_ ratios [62,63,64]. 

PRE. The PRE method allows for determining long-range distances between amino acids, typically in the range of 12–25 Å [65,66,67] (illustrated in Figure 1), compared to NOE-derived interproton distances of less than 6 Å [68]. The technique involves attaching a nitroxide spin label, such as MTSL, to a cysteine residue (either native or introduced by mutagenesis) via a disulfide bond. The spin label enhances the transverse relaxation rates of nearby amino acids, resulting in line-broadening due to dipole–dipole interactions between the spin label and NMR-active nuclei. This paramagnetic effect follows an inverse sixth power of the distance between the label and the observed residue, as demonstrated in folded proteins or complexes [69,70]. For many disordered proteins, this PRE method is beneficial in identifying long-range interactions between amino acids [71,72,73,74,75,76]. It provides information on many amino acid pairs simultaneously, compared to methods that monitor the distance between a single pair of amino acids. An interesting expansion is double spin-labeling, also referred to as paramagnetic relaxation interference [77,78,79], where the two paramagnetic sites enable accurate triangulation of individual amino acids of interest by probing the collective effect of spin labels on these amino acids without severe intensity attenuation, practically outside the intermediate surroundings of the spin labels.

Solvent-PRE. The solvent accessibility of specific residues can be probed without covalently attaching probes to the protein’s amino acids by using highly soluble paramagnetic agents such as Gadodiamide, also known as Omniscan. These agents diffuse freely and rapidly and enhance the transverse relaxation rates of nearby nuclei, such as amide hydrogens [80,81]. The rate of enhancement is proportional to the concentration of paramagnetic agents in the solution, making it an adequate measure of solvent accessibility. This method has been used to characterize folded proteins [81] and has been applied to disordered proteins showing low solvent exposure of a native-like beta-hairpin and overall high solvent exposure for the rest of the denatured ubiquitin [82,83]. Compared to non-NMR methods, this solvent-PRE method significantly improves the detection of backbone solvent accessibility with a higher amino acid coverage, as many residues are resolved by 2D NMR spectra through either proton or carton detection [84]. A new development uses two differently charged co-solutes (cationic and anionic or neutral) as free-diffusion paramagnetic agents [85,86,87]. These co-solutes are used to determine an effective per-residue electrostatic potential by utilizing an inverse sixth power of the distance between the paramagnetic co-solutes and the observed residue, particularly useful in characterizing the electrostatics of well-defined ligand-binding cavities, highly charged DNA-binding surfaces, or electrostatics-driven protein–protein interfaces.

## 3. Theoretical and Computational Biophysical Techniques

Experiments are often accompanied by theoretical and computational methods in various forms. This section explores four aspects of this collaboration between computation and experiment, arranged by ease of application. These aspects include sequence-based predictors for distinguishing IDPs from folded proteins, polymer models for interpreting experimental measurements, molecular simulations and modeling techniques that are parameterized via experimental data, and ensemble-fitting that integrates experiments and computations. 

### 3.1. Prediction from the IDP’s Primary Amino Acid Sequence 

In the 1990s, while investigating proteins involved in transcription, it was observed that the minimum requirement for functional amino acid sequences often included highly acidic contents and negatively charged amino acids [88,89]. Given the repulsive interactions involved in short regions of tens of amino acids, it was difficult to imagine these regions could fold into a well-defined three-dimensional structure, as often seen in folded proteins. Increasing numbers of sequence segments without a definable “structure” led to attempts at sequence-level classifications between disordered and structured regions of proteins. In 2000, Uversky and Dunker introduced a diagram using two sequence-based descriptors, mean net charge and mean net hydrophobicity, and found that known folded proteins and IDPs often occupy different regions of this two-dimensional diagram [90]. This work demonstrated qualitatively that the physical properties of individual amino acids could be used to predict the general structural preference of IDPs, despite some exceptions where IDPs cross the folded–disordered boundary [6].

**Figure 2:**
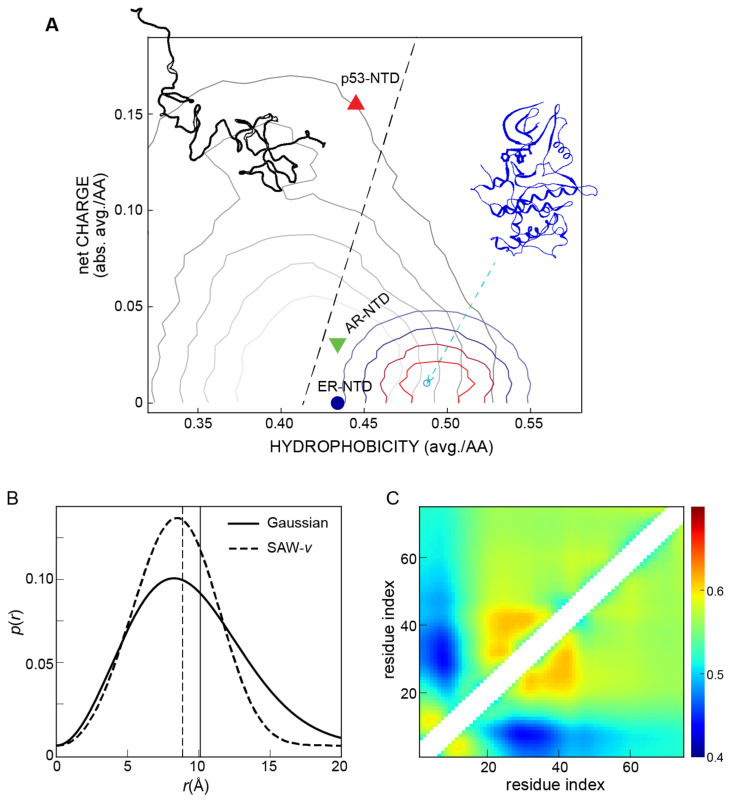
Theoretical and computational methods for studying IDPs. (**A**) Folded vs. disordered proteins in a two-dimensional plot of hydrophobicity and net charge of individual proteins. Blue: a representative folded structure of CDK7 kinase (PDB id: 7B5O [105]); colored lines: a contour plot derived from a large set of high-resolution folded protein structures available from the Protein Data Bank via the Top2018 database [106]. Black: a representative disordered structure (PDB-Dev id: PDBDEV_00000027 and SASBDB id: SASDEE2); gray lines: a contour plot derived from a set of disordered proteins via the DisProt database [104]. Dashed line: a boundary line of <*H*> = (|<*q*>| + 1.151)/2.785 as proposed by Uversky [90] where <*H*> is the average hydrophobicity per amino acid [95] and |<*q*>| is the absolute value of average net charge per amino acid. Most folded proteins are within the border of this boundary line, while disordered proteins spread over a broad range of space. Red triangle: p53-NTD (M1-V97; UniPort id: P0463; <*H*> = 0.445 and |<*q*>| = 0.155); green triangle: AR-NTD (M1-K558; UniPort id: P10275; <*H*> = 0.434 and |<*q*>| =0.031); blue circle: ER-NTD (M1-Y184; UniPort id: P03372; <*H*> = 0.434 and |<*q*>| = 0.000). (**B**) Polymer models for deriving single-distance distributions indicate that the interpretation of experimental measurement is highly model-dependent by converting a FRET efficiency of 0.2 from a 100 amino-acid peptide to the distance distribution function *p*(*r*) [107]. (**C**) Pairwise scaling exponent map from coarse-grained simulations for the N-terminal disordered region of E-cadherin [49].

Sequence descriptors. Investigations have been carried out on various amino acid properties to determine their suitability for IDP prediction. The first type of sequence descriptor focuses on charged amino acids. The fraction of charged amino acids often affects the contribution of other sequence descriptors based on charged amino acids to the overall conformational preference. For instance, IDPs with a high content of charged residues depend primarily on the arrangement of their charged amino acids [91]. When determining the overall attractive or repulsive interactions, one can look at more detailed charge-relevant sequence descriptions such as net charge per residue (NCPR) or a more complex fraction of positively/negatively charged amino acids, which are often thought to be more effective in predicting the conformational preference of an IDP [92]. The second type of sequence descriptor is based on the hydrophobicity of amino acids, and several hydrophobicity scales are available for the 20 amino acids [93,94,95]. In addition, secondary structure preference [96,97] and solvent accessibility [98] of amino acids can be used as inputs to predict disordered regions. Furthermore, a fraction of different types of amino acids can be used, and in some cases, a specific type of amino acid has been found to be important to the conformational preference of an IDP [99,100,101]. However, when considering an n-gram language model (e.g., the fraction of an n-amino-acid pattern) in a protein sequence, there may be many such sequence descriptors, and a machine learning method is often needed to achieve predictive power [102]. Figure 2A provides an example using two representative sequence descriptors introduced by Uversky [90]: the absolute value of the net charge per residue |<*q*>| and the amino acid hydrophobicity per residue <*H*> [95]. The folded proteins used here were obtained from the TOP2018 database [92] (excluding the regions that cannot be assigned a secondary structure with DSSP software [103]), while disordered proteins were obtained from the DisProt database [104] (with a criterion for a chain length of longer than 30 amino acids). As shown in Figure 2A, most folded proteins are located within the border of this Uversky-proposed boundary line, while disordered proteins occupy a broad range of space. With the increasing number of IDPs, there can be quite a few getting close to the well-folded protein regime, suggesting these proteins have similar sequence properties, at least in terms of the two sequence descriptors used.

Machine learning methods. Several machine learning methods, from simple linear regression to more sophisticated approaches such as support vector machines and deep learning (artificial neural networks with multiple layers), can combine existing sequence descriptors to predict the disordered sequences. With the increasing degrees of freedom (sequence descriptors) and increasing training datasets (e.g., DisProt [108], IDEAL [109], MobiDB [110], and solved PDB structures), deep learning has become a commonly used method for this purpose. A recent assessment testing 43 predictors found that machine learning methods and specifically deep learning methods outperform physicochemical methods [111]. However, predicting disordered regions for binding can still be challenging. Due to the ease of applying these predictors, most of which have existing web interfaces [112,113,114,115], one can always try several methods and increase the confidence level of determining the disordered region when facing a new sequence. However, the contribution of a specific sequence descriptor to the prediction or the underlying sequence grammar can often be challenging to access due to the hidden layers of deep learning methods. 

Short sequence regions. Significant efforts have been devoted to exploring short sequence regions that facilitate specific interactions between disordered regions and various biomolecules [116]. Two major categories of these regions are molecular recognition fragments (MoRFs) and short linear motifs (SLiMs). MoRF can undergo a disorder-to-order transition upon binding to their partner and can be predicted using various methods with sequence lengths ranging from 5 to 25 amino acids [117,118,119,120,121]. On the other hand, SLiMs are short sequence patches each containing 3 to 15 amino acids that are often found within the disordered regions of diverse proteins and can be highly conserved [116,122,123,124,125]. Such sequence conservation, e.g., within low-complexity disordered regions, suggests potential coevolution with binding partners for specific functions. In this case, sequence-based algorithms have been developed to predict binding regions within an IDP that interact with other proteins [126,127], nucleic acids [128], and even lipids [129]. Databases such as DIBS [130] and FuzDB [131] can be used for this purpose or as a training dataset for their algorithm development. Recent evidence has suggested coevolution between SLiMs and linkers for a particular IDP [132], indicating that flanking regions with less-conserved sequences in IDPs might also affect interactions between these short sequence regions and their binding partners [133], although this realization remains to be validated on a case-by-case basis.

Patterning of sequence descriptors. One can also investigate the patterning of existing sequence descriptors, which might provide additional physical insights. It has been shown that charge patterning, for example, plays a significant role in determining individual chain configurations [134,135,136]. By considering the patterning of even hydrophobic amino acids, often thought of as secondary to charged interactions, predictions of global properties such as polymer scaling exponent and radius of gyration are further improved [137]. Charge patterning can also be applied to understand the interactions between two IDPs dominated by the patterning of the charged amino acids [138]. More interestingly, the charge block idea has been realized for some critical IDP functions [139,140].

It should be noted that structure prediction methods such as AlphaFold v2.0 [141,142] can be used to distinguish folded and disordered regions. In addition, sequence-based algorithms can be extended to predict other factors such as prion-like domains [143,144], liquid–liquid phase separation [145,146,147], protein aggregation [148,149], and mutual synergistic protein folding [150], with an increasing number of experimental measurements serving as the training data set. A clear advantage of sequence-based predictors is their ease of use. Many predictors come with a web interface, making them accessible to quickly analyze new sequences of interest before more complex computational and experimental techniques are applied. Therefore, it is recommended to use sequence-based predictors before using any other computational/theoretical methods for IDPs. Even though there is ongoing interest in developing computational models for both folded and disordered proteins, most of the methods described here only apply to IDPs.

### 3.2. Polymer Models for Interpreting Experimental Measurements

Experimental measurements typically correspond to averaged physical variables from an ensemble of diverse conformations. Without a physics-based model, it is nontrivial to convert the experimental measurements directly. For instance, an experimental measurement that provides the distance between two amino acids still requires a distance distribution function to connect the experimental signal and the distance *r*. This can be performed through various methods, ranging from polymer models with analytical equations for *p*(*r*) to all-atom explicit solvent simulations. This section briefly describes polymer models, often the first step for interpreting experimental data.

Gaussian chain. When looking at the sizes of IDPs measured using SAXS (i.e., *R_g_*), FRET (i.e., distance *R*), and dynamic light scattering (DLS) or pulsed-field gradient NMR (i.e., hydrodynamic radius *R_h_*), IDPs of varying chain lengths *N* were found to be close to the scaling behavior of a random coil as *R_g_, R_h_ or R~N*^ν^, where ν is the scaling exponent [3,4,151]. Therefore, a Gaussian chain model [5] is often used for analyzing, e.g., FRET data and helping convert the FRET efficiency into the distance between the pair labeling positions. The distance distribution function *P*(*r*) of the model can be written as

$$P\left(r\right)={\left(\frac{3}{2\pi }\right)}^{3/2}\frac{4\pi }{R}{\left(\frac{r}{R}\right)}^{2}\exp\left[-\frac{3}{2}{\left(\frac{r}{R}\right)}^{2}\right]$$

where *R* is the root mean squared distance of all the conformations in the ensemble and *r* is the distance between a specific pair of amino acids for one conformation. Then, *R* can be obtained by minimizing $\left|\int E\left(r\right)P\left(r\right)dr-E_{expt}\right|$, where *E*(*r*) is the Förster equation [47] describing the FRET efficiency as a function of the distance and *E_expt_* as the experimentally measured FRET efficiency. However, for one specific IDP, the scaling exponent can differ from 0.5. A FRET investigation that labeled multiple pair positions on different proteins revealed that six IDPs exhibit scaling exponents between approximately 0.45 and 0.65 [10]. It has been noted that the Gaussian chain model tends to overestimate the *R* value interpreted from FRET efficiency when the specific IDP is closer in behavior to an excluded volume chain [152].

Self-avoiding walk. A more general polymer model other than the Gaussian chain model is the self-avoiding walk (a polymer which cannot cross itself) model, in which the distance distribution *P*(*r*) can be adjusted according to the scaling exponent, referred to as a SAW-ν model, and the *P*(*r*) can be written as

$$P\left(r,\nu \right)=A\frac{4\pi }{R}{\left(\frac{r}{R}\right)}^{2+g}\exp\left[-B{\left(\frac{r}{R}\right)}^{\delta }\right]$$

where *R* is the root mean squared distance, A and B are obtained from the conditions $1=\int_{0}^{\infty }P\left(r\right)dr$ and $R^{2}=\int_{0}^{\infty }r^{2}P\left(r\right)dr$, and the exponents are given by g≈(γ−1)/ν [153], γ = 1.1615 [154], and $\delta =\frac{1}{1-\nu }$ [155]. Then the scaling exponent *ν* can be obtained by minimizing $\left|\int E\left(r\right)P\left(r,\nu \right)dr-E_{expt}\right|$ by the restraint of $R\propto N^{\nu }$. We show in Figure 2B, with provided FRET efficiency of 0.2 of a 100 amino-acid peptide, that the *P*(*r*) reconstructed using a Gaussian chain and the SAW-ν model can be quite different, suggesting such data analysis is model-dependent. The results from the SAW-ν model have been in close agreement with the all-atom explicit solvent simulations [107], which are usually computationally demanding to generate. The SAW-ν model can also be applied to other experimental methods to provide the distance between two specific amino acids. For instance, for PET and PRE, one can easily replace the *E*(*r*) in the previous minimization with the equation corresponding to the experimental signal and the distance, and then the *P*(*r*) can be obtained using a different experimental method. In addition, methods that provide *R_g_* from SAXS data can be compared with the methods that provide distance *R* via the relation between *R_g_* and *R* [156],

$$\lambda =\frac{R^{2}}{R_{g}^{2}}=\frac{2(\gamma +2\nu )(\gamma +2\nu +1)}{\gamma (\gamma +1)}$$

where *γ* = 1.1615 [154]. SAXS can be analyzed similarly with a higher-order correction factor to the original Guinier analysis [157].

Another advantage of using the SAW-ν model is that it provides the scaling exponent in addition to *R*. The scaling exponent sometimes tells more than just the size of an IDP. For instance, in the case of liquid–liquid phase separation, a strong correlation was found between the critical temperature of phase separation and the theta-solvent temperature at which the scaling exponent is 0.5 [158]. It is important to note that the scaling exponent is only well-defined for a homopolymer, and the polymer models discussed assume that the IDP being studied is a homopolymer. This assumption is acceptable for some IDPs with low-complexity sequences and weak nonspecific interactions. However, growing evidence suggests specific IDPs exhibit transient interactions between pairs of amino acids [159,160]. Further work may be required, such as incorporating a new term into the current polymer model or using more sophisticated simulation models.

### 3.3. Molecular Simulations and Modeling Methods

Computational simulation and modeling techniques require experimental data for parameterization and calibration and can be computationally demanding. However, once established, these techniques can be applied to a wide range of systems beyond those used for parameterization. Techniques include all-atom explicit or implicit solvent simulations and coarse-grained modeling, which differ in the level of detail they provide for amino acids and water molecules. Choosing the proper simulation method requires finding a balance between detail and feasibility. All simulations rely on experimental data for parameterization or validation of results. Low-resolution models typically have fewer free parameters and require less experimental input. This approach may be appealing due to their physical intuition for understanding the underlying mechanisms. However, they may lack the detail to capture experimental measurements accurately. Higher-resolution models have more free parameters and thus require more experimental data, but they may not be easily transferable to new proteins without verification. Other approaches, such as Rosetta [161] and AlphaFold [142], can be used to model disordered regions that may be partially structured but not discussed here.

All-atom simulations. All-atom explicit solvent simulations offer the highest resolution and may be able to describe specific residue interactions that can be lost in lower-resolution coarse-grained models, such as hydrogen bonds, salt bridge, cation-π, sp2/π interactions, and general hydrophobic/van der Waals interactions [162]. Since water molecules are explicitly represented, all-atom explicit solvent simulations can also lead naturally into discussing the dynamics of IDPs rather than just the averaging equilibrium properties [163]. For instance, end-to-end chain relaxation time from all-atom simulations have been found in close agreement with that estimated from the FRET experiment [164]. However, one major challenge of using all-atom explicit solvent simulations is the accuracy of the force field [165,166,167,168,169]. Since an IDP lacks a nonlocal tertiary structure, this enlarges minor inaccuracies of local secondary structure preference and amino acid interactions of old force fields. Recent attempts to improve the accuracy of all-atom force fields rely primarily on implementing better dihedral potentials for reproducing secondary structure propensities [170] and fine-tuning protein–solvent interactions for capturing the sizes of IDPs [165]. Another option is to use implicit instead of explicit solvent [171]. Implicit solvent can be problematic when simulating interactions between charged amino acids at physiological ionic strength. This issue can be solved by introducing explicit ions such as the ABSINTH model [172,173], which can be a good alternative between an all-atom explicit solvent model and more reduced coarse-grained models.

However, all-atom models are challenging for simulating more complex phenomena with more than one chain in the simulation, such as folding upon binding [174], liquid–liquid phase separation [162], or aggregation [175] due to the high computational cost for obtaining trajectories with sufficient time scales. There can be a few possible ways to overcome sampling difficulties. One option is the use of advanced sampling methods. Replica exchange molecular dynamics (REMD) can be applied to IDPs [176,177] and have been applied to the p53 disordered region [178]. Despite the high computational demands, this REMD method can be combined with other advanced sampling techniques, such as Gaussian-accelerated molecular dynamics (GaMD) [179,180], to enhance its capabilities further. Combining REMD and GaMD has been applied for the ER disordered region [6]. Collective-variable-based methods are commonly applied to folded proteins [181,182,183]; however, their application to IDPs remains to be seen due to the lack of obvious collective variables for IDP dynamics. Other approaches to accelerate simulations include using specialized supercomputers such as Anton [184] or implementing GPU-assisted versions of molecular dynamics packages [185,186,187]. 

Coarse-grained simulations. Coarse-grained (CG) models further reduce the complexity of amino acids in addition to just implicit solvents. Resolution varies greatly across CG models according to their intended use, from several CG beads for each residue (e.g., AWSEM [188,189,190], and flexible-meccano [191]), to one CG bead per residue, to one CG bead for the entire domain. One needs to choose an appropriate resolution depending on the problem of interest. A model with a resolution of one bead per residue could be a good balance between reducing computational cost and achieving amino acid specificity. Here we briefly describe one example, the HPS model [192]. There are three different types of interactions of local bonded interactions, electrostatics, and short-range pairwise interactions. The electrostatic interactions are modeled using a Coulombic term with Debye-Hückle electrostatic screening [193] to account for salt concentrations. The short-range pairwise interactions account for protein–protein and protein–solvent interactions and are based on the amino acid hydropathy scale [99]. In the current HPS model, the Ashbaugh–Hatch functional form is used for the short-range pairwise interactions [194], but other functional forms can be used to consider the nonbonded interactions in addition to the electrostatic interactions between charged amino acids [195,196,197,198]. The overall interaction strength of this pairwise interaction term and amino-acid-specific parameters (e.g., hydropathy scales) can be optimized with the experimental data of IDPs [190,199,200,201]. This term can also be temperature-dependent on accounting for the upper and lower critical solution temperatures [202] and salt-dependent to account for the salting-out effect at high salt concentrations [203]. Additional angle and dihedral terms can be introduced to capture the residue-specific secondary structure propensities of the chain [204,205]. The entire framework is flexible and easy to re-optimize with growing experimental measurements [190,199,200,201] and can be extended to biomolecules such as nucleic acids [206]. The HPS model lacks specific interactions such as hydrogen bond, salt bridge, cation-π, and sp2/π interactions and often underestimates specific strong interactions that might exist in a particular IDP. However, this model is usually sufficient to capture interactions between charged amino acids. As shown in Figure 2C, the HPS model can correctly capture the attractive interactions (blue in the scaling exponent map) between charged amino acids within the N-terminal region of the disordered E-cadherin protein. These interactions lead to salt-induced expansion of the first 40 amino acids in contrast to the salt-induced collapse of the other regions of the protein seen in the FRET measurement. 

### 3.4. Computational Strategies for Combining Multiple Experimental Measurements

**Figure 3:**
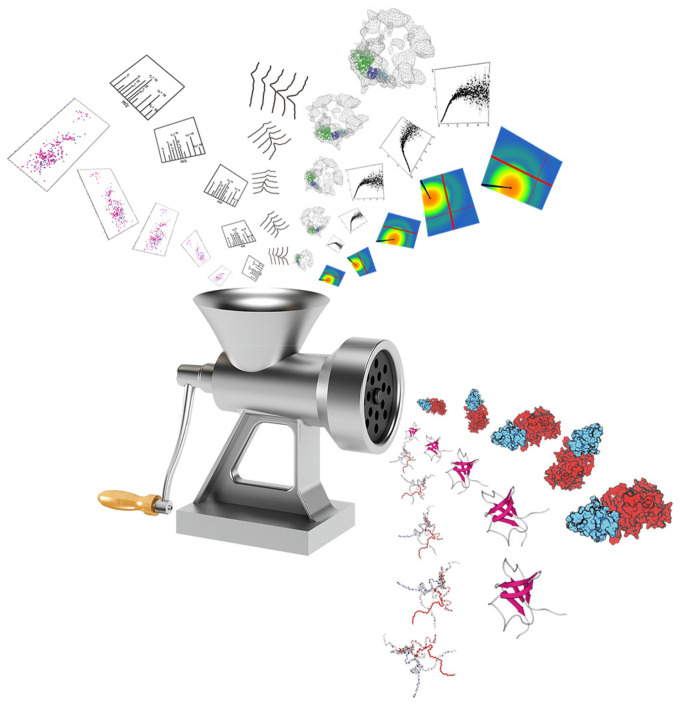
A schematic diagram for integrative biophysics combining various biophysical datasets. Complementary restraints from experimental studies of small-angle X-ray scattering, site-specific solvent accessibility, and various NMR techniques, as well as computations, are fed into ensemble-fitting machinery to generate a comprehensive picture for the ensemble structures of highly flexible biomolecules such as intrinsically disordered proteins. Reprinted with permission from Ref. [215].

Simulation models that are parameterized with experimental data are often considered transferable. However, when applied to a new system of interest, they may not always match the latest experimental data, requiring further improvement. Re-optimizing the model with new experimental data is a straightforward solution, but this is often performed with CG models due to fewer built-in free parameters. Optimizing all-atom models to match a new set of experimental data can be time-consuming. Nonetheless, two alternatives include biased simulations with experimental data as restraints [207,208] and ensemble fitting that reweights the conformations of existing ensembles to best fit experimental data [152,209,210,211,212,213,214]. As integrative biophysics approaches are emerging [215], both methods are critical for IDP characterization by integrating these various experimental inputs, as depicted in Figure 3. 

Central to integrative biophysics is the development of “bridges” and “connectors” between computations and experiments. These tools allow experimental measurements to be calculated from the conformations of IDPs obtained using computational methods. Linking to SAXS data includes model-free coarse-grained computing [216,217,218] and atomistic-level modeling [219,220]. HDX and HRPF analysis mainly utilize protection factor analyses [18,41] to connect the solvent accessibility surface area. For NMR measurements, PRE data can be analyzed via ensemble averaging over inverse sixth power of distances [72], while solvent-PRE data can be analyzed via grid-based surface volume calculations [81,221,222]. FRET efficiencies can be calculated from a distance between the two labels using the Förster equation [47], and PET rates can be estimated using an exponential function to the distance through all-atom modeling [53]. DEER spectra are typically converted to distance distributions before being applied to computational methods [50,223,224,225]. Such tools play a critical role in advancing integrative data analysis.

Experiment-restrained simulations. Experiment-restrained simulation methods have succeeded in exploring new conformations or leading simulations toward conformational changes of interest. However, unlike folded proteins, these methods are often not straightforward for IDPs. IDP measurements are the results of ensemble-averaged features, making it difficult to determine how to design the biasing potential for simulations. This ambiguity makes biased IDP simulations rely on the time-consuming processes of replica averaging [208], maximizing entropy, and extensive iterations [226,227,228,229]. Examples include modeling strategies with experimental restraints from SAXS [230,231], DEER [232], HRPF [233], FRET [234], and NMR observables [207,208,235,236]. Additionally, the overall results are influenced by the accuracy of the physics-based model used. Improvements in all-atom force fields and growing sources of experimental data are expected to alleviate some of these concerns.

Ensemble fitting. As an alternative approach, ensemble fitting directly incorporates experimental bias into ranking and scoring candidate structures obtained from computations. This ensemble approach is achieved by post-processing an ensemble of these putative conformations, where weights are assigned to individual conformations and then adjusted to best fit experimental observables. One prolific example of ensemble fitting is the combination of SAXS data with various docking and modeling algorithms. This SAXS-assisted method has been implemented and applied to various research areas, including protein–protein interactions [237,238,239,240,241,242,243], high-order structures [213,214,244,245,246,247], protein dynamics [248,249,250,251,252,253,254,255,256,257], RNA dynamics [258,259,260,261], and the study of IDPs [6,262,263,264,265]. 

Ensemble fitting is frequently combined with multiple experimental data types to obtain a complete picture of protein behavior. By combining SAXS or FRET data about global conformations with site-specific information on solvent accessibility (e.g., HRPF and DFCS) or NMR distance data (e.g., PRE), insight has been gained into the behavior of IDPs [72,212,214,264,266,267,268,269]. As shown in our recent publication [42], the amino acid contact map of the ER disordered domain can be obtained through ensemble fitting of data from SAXS, HRPF, and DFCS, which reveals previously unknown nonlocal contacts. 

It is still an open question regarding how to best proceed with ensemble fitting to meet all experimental measurements. Two different strategies have been employed to prepare the basis set of initial conformations for fitting. One involves using a large pool of candidate structures for maximum entropy analysis [270], while the other requires minimizing the number of conformational clusters before fitting [250]. The first strategy involves handling a large number of structures and applying the maximum entropy principle to prevent overfitting, recognizing that some structures share similar experimental observables. The second strategy conducts conformational clustering before fitting and requires well-defined collective variables that can separate the pool of structures into distinct clusters, serving as a basis set of conformations for ensemble fitting. A combined approach has been attempted using a modest number of conformations and the maximum entropy method [6,271] that has successfully made predictions that were subsequently validated. If the initial pool of structures captures the majority of target conformations, then conformational clustering based on experimental observables before fitting could be considered a method of choice instead of imposing a statistical bias on the fly, where a minimum number of distinct conformations is utilized as a de facto basis set to avoid a potential issue of double-counting in ensemble fitting (i.e., using both a non-equal probability weight for individual conformations and an entropy penalty for the overall density of conformations, simultaneously). Nevertheless, this assertion requires further investigation in future studies.

## 4. Targeting Protein Intrinsic Disorder as a New Frontier of Drug Discovery 

**Figure 4:**
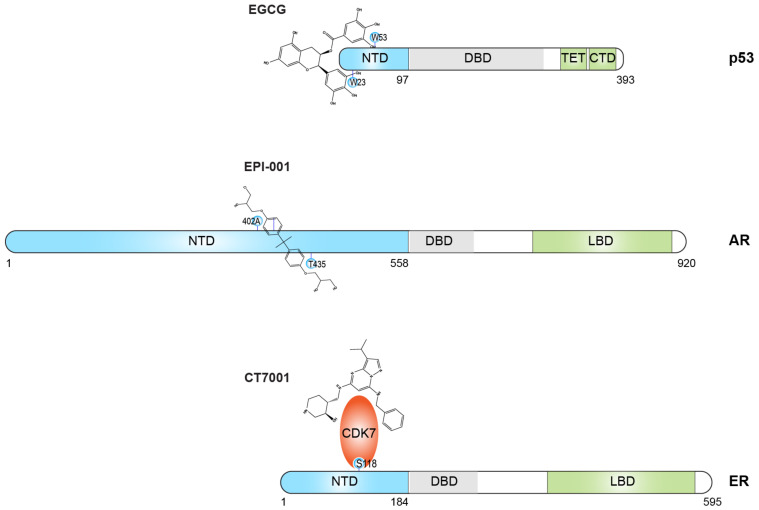
Small molecule targeting against protein intrinsic disorder as a new frontier in drug discovery. NTD: N-terminal domain; DBD: DNA-binding domain; LBD: ligand-binding domain; TET: tetramerization domain; CTD: C-terminal domain. AR: androgen receptor; ER: estrogen receptor. EGCG, a compound found in green tea, has been shown to interact with two specific amino acids of p53-NTD, W23 and W53 [276]; EPI-001, isolated from marine sponges [283], has been found to interact with A402 and T435 residues of AR-NTD [284]. CT7001 has been demonstrated to bind the ATP-binding site of CDK7 (PDB id: 7B5O [91] and 6Z4X [285]), a serine/threonine kinase that can phosphorylate Ser118 within the ER-NTD [286].

IDPs are emerging as a promising class of targets for small molecule binding [272,273,274]. Notable examples of these ligands include 10058-F4/sAJM589 targeting the transcription factor c-Myc [275], EGCG against p53-NTD [276], Fasudil against α-synuclein [277], 10074-G5 against Aβ42 [278], SJ403 against p27-Kip1 [279], EPI against AR-NTD [280], CLR01 as a molecular tweezer against the disordered protein–protein interface of Cdc25C [281], and NSC635437 against the fusion oncoprotein EWS-FLI1 [282], some of which are depicted in Figure 4.

p53-NTD has been extensively studied using various biophysical techniques, including SAXS [287], PRE [288], solvent-PRE [84], and PET coupled with fluorescence correlation spectroscopy [54], as well as computations [178,287]. Knowledge accumulated over the years has not only aided in understanding the binding mechanism between EGCG and p53-NTD [276], but also provided the molecular basis for finding new binders.

AR-NTD was among the first IDPs selected as a therapeutic target for drug development [280,289]. Unlike p53-NTD, small molecule binders were identified before biophysical data of AR-NTD binding became available. EPI-001, one of the early compounds, was isolated from marine sponges and found to inhibit AR-NTD transcriptional activity [283,290]. Subsequent binding characterization included chemical shift perturbation analysis [288] as well as computational modeling [291]. Despite the wealth of biophysical data available for p53-NTD, a comprehensive structural ensemble of p53-NTD (either in the absence or presence of EGCG) is not currently available that explicitly accounts for the diverse experimental restraints. Encouragingly, chemical shift perturbations have identified specific amino acids that are well separated in their primary amino acids for ligand–protein interactions, as indicated in Figure 4.

Studies targeting ER-NTD have lagged behind AR-NTD, and there is currently no small molecule inhibitor that directly binds ER-NTD. While ER-NTD and AR-NTD belong to the same nuclear receptor superfamily, ER-NTD is shorter (184 amino acids) than AR-NTD (558 amino acids) [292]. Despite being shorter than AR-NTD, it is longer than p53-NTD (97 amino acids) [293], as illustrated in Figure 4. Counterintuitively, the structural information for ER-NTD is limited [294,295] compared to AR-NTD and p53-NTD, whose chemical shifts have been mostly assigned. Maintaining protein stability and homogeneity has posed difficulties in conducting NMR studies on ER-NTD. However, non-NMR studies have provided early insights into its inner workings, including SAXS, HRPF, and DFCS data and computational studies (5, 28).

While no small molecule directly targets ER-NTD, efforts are underway to develop small molecule inhibitors that target its coregulatory proteins. As illustrated in Figure 4, CDK7 is an upstream protein kinase that activates ER-NTD by phosphorylating serine at position 118 [286], and CDK7 inhibitors have been developed to reduce ER-NTD activity [296,297]. One such inhibitor, CT7001, is currently undergoing clinical trials for the therapeutics of ER-positive breast cancer (phase 2) and castrate-resistant prostate cancer (phase 1 as of February 2023) [298]. However, the multifaceted role of CDK7 as an activation initiator for multiple proteins involved in transcription and cell cycle regulation can lead to cellular toxicity and off-target effects. As we gain more knowledge about ER-NTD at the molecular level, a more direct strategy is approaching to target ER-NTD for the discovery of novel binders.

These examples represent one approach of targeting protein intrinsic disorder at specific protein regions or post-translational modifications to shift the equilibrium of disordered conformations. Another strategy involves using small molecules or peptides that mimic binding partner proteins to alter the disordered protein–protein interface [299,300,301,302]. Notably, a significant portion of such protein–protein interactions is mediated by so-called short linear motifs (SLiMs), commonly found within disordered regions [303]. Typically, each SLiM is a small polypeptide stretch consisting of 3 to 15 residues [122,125] and can be grouped into six distinctive classes via the eukaryotic linear motif (ELM) database [304,305], including the LIG class for covering the function of protein interactions with ligand proteins, MOD for post-translational modifications such as phosphorylation, CLV for proteolytic cleavage, TRG for subcellular targeting, DEG for degradation with protein polyubiquitylation, and DOC for classic docking of enzyme recruitment [306]. The classification of SLiMs into distinct classes provides a comprehensive understanding of the multifaceted functions that many SLiMs can carry out, even within the same disordered protein sequence. For instance, the discrimination between LIG and MOD classes is an excellent example of how protein phosphorylation sites are distinct from protein–protein interaction sites; the fact that phosphorylation sites are categorized as MOD motifs and not LIG motifs indicates that they may not directly participate in protein–protein interactions. This distinction has been demonstrated through the analysis of ELM search results of, e.g., p53-NTD, AR-NTD, and ER-NTD, where their SLiMs spread over the amino acid sequence with little overlap. Given that IDPs often engage in promiscuous interactions with a vast array of partner proteins [1,307], a thorough investigation of the IDP of interest is important to identify whether a particular SLiM dominates over or coordinates with others in order to fully understand its potential as a drug target. 

## 5. Perspectives: Chaotic Life of Protein Intrinsic Disorder at a Crossroads 

Intrinsic disorder in proteins imposes difficulties for biophysical studies and challenges the conventional structure–function paradigm learned from folded proteins. As IDPs are critical in many biological processes, such as transcription and signaling, understanding the inner workings of IDPs requires innovative use of available biophysical tools and a proactive approach combining complementary techniques. The limited yet growing knowledge provides a new perspective on the IDPs’ sequence–structure–function relationship, thereby allowing for the study of protein intrinsic disorder to find new binders against important therapeutic targets.

## Data Availability

Not applicable.
